# An Approach to Evaluate Blurriness in Retinal Images with Vitreous Opacity for Cataract Diagnosis

**DOI:** 10.1155/2017/5645498

**Published:** 2017-04-26

**Authors:** Li Xiong, Huiqi Li, Liang Xu

**Affiliations:** ^1^School of Information and Electronics, Beijing Institute of Technology, Beijing 100081, China; ^2^Beijing Institute of Ophthalmology, Beijing Tongren Hospital, Beijing 100730, China

## Abstract

Cataract is one of the leading causes of blindness in the world's population. A method to evaluate blurriness for cataract diagnosis in retinal images with vitreous opacity is proposed in this paper. Three types of features are extracted, which include pixel number of visible structures, mean contrast between vessels and background, and local standard deviation. To avoid the wrong detection of vitreous opacity as retinal structures, a morphological method is proposed to detect and remove such lesions from retinal visible structure segmentation. Based on the extracted features, a decision tree is trained to classify retinal images into five grades of blurriness. The proposed approach was tested using 1355 clinical retinal images, and the accuracies of two-class classification and five-grade grading compared with that of manual grading are 92.8% and 81.1%, respectively. The kappa value between automatic grading and manual grading is 0.74 in five-grade grading, in which both variance and *P* value are less than 0.001. Experimental results show that the grading difference between automatic grading and manual grading is all within 1 grade, which is much improvement compared with that of other available methods. The proposed grading method provides a universal measure of cataract severity and can facilitate the decision of cataract surgery.

## 1. Introduction

A cataract occurs when there is a buildup of protein in the lens. The protein buildup makes lens cloudy; thus, some of the light is prevented from passing through the lens and vision is impaired [1]. This is mainly why cataract is the principle cause of partial to complete blindness. It is reported that cataract patients account for almost half of global blindness [2]. Research also suggests that the number of people worldwide who have lost their vision is increasing by approximately 1 million per year and this number is expected to increase up to 40 million by the year 2025 [3]. Although surgery is an effective treatment available for these patients. An accurate diagnosis of the severity of cataract is still needed prior to any surgical interventions to ensure both safety of the patients and high-quality treatment.

Cataracts are classified into three types according to the location of opacity: nuclear cataract, cortical cataract, and posterior subcapsular cataract. Clinically, nuclear cataract is diagnosed using slit-lamp photography, while cortical and posterior subcapsular cataracts are diagnosed using retroillumination imaging devices. Clinical grading of cataract is performed via comparing the observed picture with a set of standard photos with different cataract severities. Lens Opacities Classification System III (LOCS III) [4] is widely applied in clinical diagnosis. Besides, the Oxford Clinical Cataract Classification [5] and the American Cooperative Cataract Research Group (CCRG) method [6] are similar grading criteria. The criteria mentioned above require different image modalities to diagnose cataract which can be graded separately for each individual type. A universal evaluation of cataract severity is essential to determine the timing of cataract surgery and the priority of surgery when surgery service is not sufficient.

Since the first fundus camera was invented in 1910 [7], the retinal image acquired by the fundus camera has been widely applied in diagnosis of ocular diseases such as glaucoma, age-related macular degeneration, and diabetic retinopathy [8]. Recently, retinal image is proposed as a new means for cataract diagnosis according to its blurriness [9]. Compared with that of slit-lamp images and retroillumination images, only one image modality is required for cataract diagnosis using retinal images, and a universal grading of cataract severity can be assessed. Two distinguished benefits are found in this method which include compatibility for telediagnosis and integration with screening of glaucoma and diabetic retinopathy. Therefore, we believe screening for cataracts using retinal images can be an efficient means not only for addressing healthcare shortage problem in developing countries via telemedicine but also for integrated screening of different retinal diseases.

**Figure 1:**
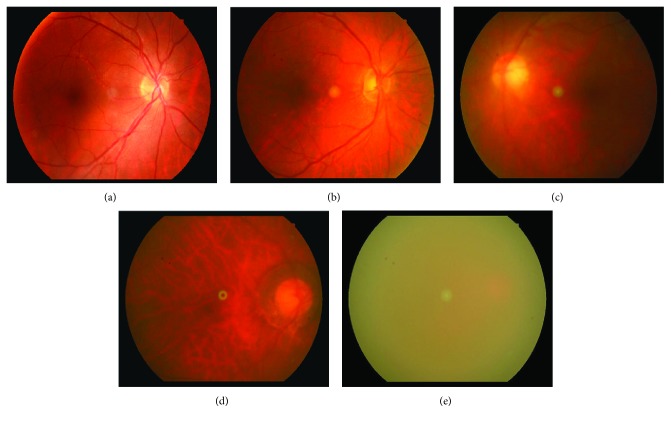
Examples of retinal images with different blurriness grades: (a) grade 0; (b) grade 1; (c) grade 2; (d) grade 3; (e) grade 4.

Opacity of lens will lead to blur photography of the retina. According to blurriness shown in a retinal image, blur level of retinal images can be classified into five grades [10].  Figure 1 shows examples of retinal images with five different grades.  Figure 1(a) shows a healthy retinal image with grade 0. In an image with grade 0, the retinal vessels (both the main vessels and capillary vessels) and optic disc can be clearly seen. In an image with grade 1, main vessels are visible while the capillary vessels are only faintly visible as shown in Figure 1(b).  Figure 1(c) presents an image with grade 2 in which small vessels are invisible and main vessels are faintly visible. In an image with grade 3, only the optic disc and vessels around the optic disc can be seen indistinctly, which is illustrated in Figure 1(d). As Figure 1(e) shows, no retinal structures can be observed in an image with grade 4. Ophthalmologists define grade 0 as noncataract and grades 1–4 as cataract cases, while no surgery is needed for grades 0-1 and timely surgery is necessary for grades 2–4 [11].

**Figure 2:**
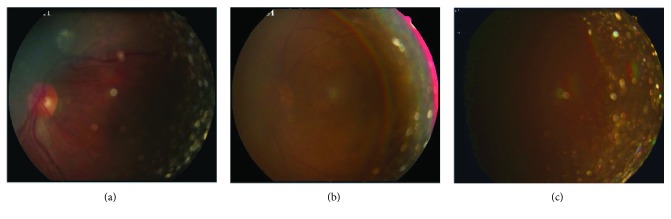
Examples of images with vitreous opacity: (a) image with blurriness of grade 2; (b) image with blurriness of grade 3; (c) image with blurriness of grade 4.

Screening for cataract using retinal images according to blurriness will be influenced by two factors: vitreous opacity and the small pupils of patients. Vitreous opacity is a common abnormality which can be observed in a retinal image. Normal vitreous body is a transparent and extracellular gel, which contains a complicated structural framework of collagen, soluble proteins, and hyaluronic acid and a water content of 99% [12]. There are many types of vitreous opacity with different causes, and they usually appear as a circular-shaped, translucent bright area in a retinal image.  Figure 2 shows some examples of a retinal image with vitreous opacity. Figures 2(a), 2(b), and 2(c) are diagnosed as cataract in grades 2, 3, and 4, respectively. It can be seen that vitreous opacity may emerge in various shapes, sizes, intensities, and positions and they are obvious even in blurry images, which will influence the detection of retinal structures and evaluation of blurriness. The small pupil of patients will also cause blur of retinal images, but it mostly affects the peripheral regions of the retinal images. Generally, the retinal images of patients will be photographed with the mode of the small pupil.

In developing countries, due to the regional economic gap, well-experienced ophthalmologists are not sufficient in some areas [13]. This fact impedes timely treatment for patients. At present, screening of cataract and other retinal diseases in these developing areas with insufficient ophthalmologists can be performed through remote consultation. Computer-aided analysis and a screening system can realize feature-based visualization and provide references for ophthalmologists, which are applicable to remote consultation. Moreover, there is difference of diagnosis between different ophthalmologists. An automatic screening system can quantify features, which is beneficial to unify diagnostic criteria.

Automatic analysis of retinal image has been investigated for many years. Most research focuses on automatic detection of retinal structures such as the optic disc [14–17], vessels [18–20], and fovea [21–23]. Based on anatomical structure detection, features of each fundus structure are extracted to detect pathological changes. Extensive studies have been performed on computer-aided diagnosis systems for ocular diseases such as microaneurysms [24], glaucoma [25–27], macular degeneration [28, 29], and diabetic retinopathy [30, 31].

Research efforts have been put into diagnosis of a specific type of cataract automatically using slit-lamp images or retroillumination images [32–34]. With retinal image becoming more and more widely used in clinical applications, automatic grading of cataract based on retinal image is proposed in recent years. Fourier analysis was proposed to estimate cataract severity in digital retinal images [35]. It showed that Fourier value has a close correlation with the combined LOCS III score and logMAR visual acuity. In this study, the detection between cataract and noncataract was evaluated, but automatic grading of cataract severity was not performed. Top-bottom-hat transformation and a trilateral filter were applied in paper [36] to extract features which include luminance feature, gray co-occurrence matrix, and gray-gradient co-occurrence matrix. Based on these features, BP neural network classifier was utilized to grade cataract severity. The wavelet transform and sketch-based methods were investigated for feature extraction in [13]. Retinal images were classified into noncataract, mild, moderate, and severe grade with multiclass discriminant analysis algorithm. The method in [37] combined methods proposed in [13, 36]. The ensemble methods, majority voting, and stacking are investigated to combine the multiple base learning models for final cataract classification.

Although cataract classification using retinal image has been investigated in the previous studies, there are still two challenges. One issue is that existing methods cannot handle the retinal images with vitreous opacity well. All available methods of screening cataract using retinal images are only based on the features of contrast between retinal structures and background [13, 35–37] without considering the influence of vitreous opacity. In these methods, vitreous opacities with strong contrast will be detected as retinal structures, which will lead to incorrect grading. Another challenge is that the performance of automatic grading needs to be improved. For example, there is still great grading difference (>1 grade) between automatic grading and clinical grading using available methods, which will hinder the clinical usage of automatic grading.

In this paper, an approach to evaluate blurriness for cataract diagnosis with vitreous opacity removal is proposed. The main contribution of this work can be summarized as follows: (1) A morphological method with multithresholds is proposed to detect vitreous opacity. The detected vitreous opacity is removed from retinal structure detection to improve the accuracy. This method is also applicable to eliminate exudates from visible retinal structure segmentation. (2) The designed approach is applicable to images with different resolutions. (3) Our grading difference between automatic grading and clinical grading is all within 1 grade, which shows that the proposed method outperforms the other existing methods. (4) Our approach is evaluated using a large amount of retinal images (>1000) with different resolutions.

## 2. Method

**Figure 3:**
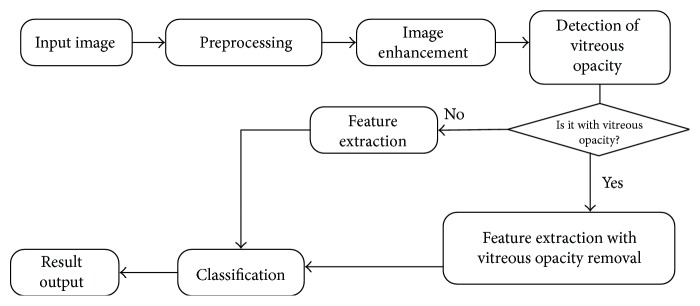
Flow diagram of the proposed cataract grading system.

The proposed automatic approach for cataract grading includes the following steps: preprocessing for image-size normalization, image enhancement, feature extraction, and cataract grading. The entire process is illustrated in Figure 3. In order to process retinal images with different resolutions, image-size normalization based on the diameter of the acquired region of interest (ROI) is performed in preprocessing. Images are further enhanced with 2D reverse Gaussian-matched filter after preprocessing, and three types of features are extracted. A decision tree is trained to classify every two adjacent grades into one class before the final grading. Pathological changes may appear in a retinal image, and these pathological changes will affect accurate feature extraction. Vitreous opacity is one type of pathological change, which is commonly presented in retinal images of cataract patients. To avoid its disturbance, a morphological method with multithresholds is proposed to detect vitreous opacity in this paper and vitreous opacity is further removed from the retinal structure segmentation.

### 2.1. Preprocessing

**Figure 4:**
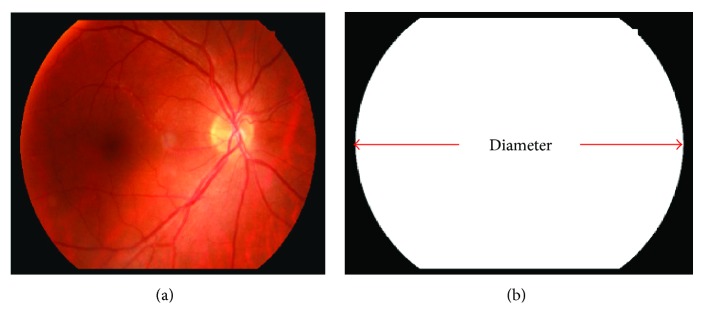
ROI mask generation: (a) original image; (b) ROI mask.

In order to handle the images with different resolutions, every image will be adjusted to the same size automatically. It should be noted that the diameter of an ROI (region of interest) mask is normalized rather than the size of an input image. To obtain this diameter, an ROI mask is obtained. Firstly, a retinal image is set at a threshold in the red channel. This method is essentially employed to separate the dark background and the retina. To avoid misclassifying areas with low illumination in the retina as background, the threshold value is set to 10, which is an empirical value. Then, morphological operators of opening, closing, and erosion are applied on the image obtained in the previous step in turn with a 3 × 3 square kernel [16].  Figure 4(b) shows an example of the generated ROI mask of Figure 4(a). When processing a new retinal image, the ROI mask is extracted and the maximal width in the horizontal direction is computed as the diameter of the fundus as shown in Figure 4(b). If the diameter is not equal to a given diameter, then the input image will be resized according to the ratio between the estimated diameter and the given diameter by bilinear interpolation. In our test, the given diameter is set to 2161 pixels. To reduce the detection error near the boundary, the ROI mask will be shrunk by 50 pixels. Next, all the parameters are set according to the size of retinal structures in normalized images. In the normalized images, the average diameter of the optic disc is about 200 pixels, and the width of the vessels are in the range of 24∼36 pixels.

### 2.2. Image Enhancement with the 2D Gaussian-Matched Filter

In order to enhance the retinal vessels and boundary of the optic disc, the 2D Gaussian-matched filter [18] is applied on green channel followed by initial processing. It is observed that retinal blood vessels usually appear darker than their surroundings and can be approximated as piecewise linear segments; we assume that the model of a vessel segment is a reverse Gaussian function. So, a Gaussian filter is employed as the matched filter. Mathematically, a Gaussian filter can be described as follows:

(1) $$\begin{array}{c} K_{i}\left(x,y\right)=-exp\left(\frac{-x^{2}}{2\sigma^{2}}\right),\left|x\right|\leq 3\sigma ,\left|y\right|\leq \frac{L}{2}, \end{array}$$

where *L* is the length of a vessel segment on a fixed orientation. The orientation of *L* is initialized along *y*-axis. *σ* represents the scale of this filter, which is related to the width of the vessel. The mean value of the filter is determined as (2), and *A* is the number of points in this filter.

(2) $$\begin{array}{c} u_{i}=\underset{\left(x,y\right)\in N}{\sum }\frac{K_{i}\left(x,y\right)}{A}. \end{array}$$

The final convolutional filter is defined as follows:

(3) $$\begin{array}{c} K_{i}^{\prime }=K_{i}\left(x,y\right)-u_{i}. \end{array}$$

In order to detect the vessel in different directions, 12 filters on 12 different orientations are generated using the rotation matrix mentioned as follows:

(4) $$\begin{array}{c} R=\left[\begin{array}{ll} \cos\theta_{i} & -\sin\theta_{i}\\ \sin\theta_{i} & \cos\theta_{i} \end{array}\right], \end{array}$$

where *θ*_*i*_ is the orientation of the *i*th filter which increases 15° from *i*th filter. Twelve filters are applied on each pixel in the green channel. For every pixel, only the maximum response among the twelve filters is retained.

**Figure 5:**
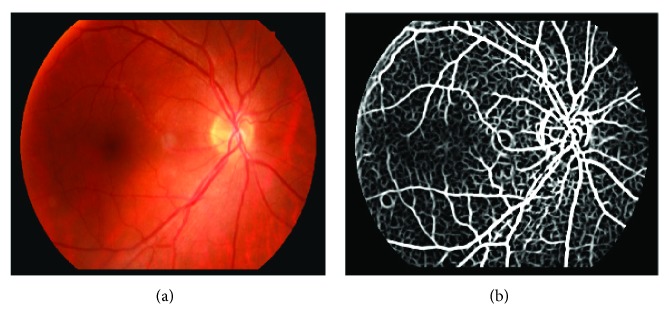
The enhancement with 2D Gaussian filter convolution: (a) original image; (b) enhanced image.

Filter size and parameter *σ* and *L* are all determined according to the vessel width of an input image. It has been proved in [18] that it is proper to set *σ* and *L* to 2 and 9 pixels, respectively, in their database, and the filter size is 15 × 16 pixels. In our paper, the size of our normalized image is 4 times of the size of images in [18]. Therefore, the filter is designed with size 60 × 61, and *σ* and *L* are set to 8 and 36, respectively. An example of the enhanced image is shown in Figure 5(b). It can be seen that the retinal vessels and boundary of the optic disc are enhanced in the retinal image.

### 2.3. Detection of Vitreous Opacity

Vitreous opacity often appears in the retinal image, which will interfere the grading of blurriness for cataract diagnosing. Due to the higher intensity compared with background, boundaries of vitreous opacity will be enhanced by the 2D Gaussian-matched filter as well. When a large area of vitreous opacity appears, the accuracy of grading will be seriously influenced because the boundaries of vitreous opacity are wrongly extracted as visible retinal structures.

To eliminate the detection error brought by vitreous opacity, a set of morphology operations are applied on the green channel of input images to enhance regions of vitreous opacity. To avoid the noises, the green channel is smoothed by a median filter at first. The size of this median filter should be less than the width of the vessels. In our paper, the size is set to 12 × 12 pixels. Close operations are performed with circle structuring elements *e*_1_ and *e*_2_ which are defined by the following:

(5) $$\begin{array}{c} d_{1}=\left(G⊕e_{1}\right)⊖e_{1}, \end{array}$$

(6) $$\begin{array}{c} d_{2}=\left(G⊕e_{2}\right)⊖e_{2}, \end{array}$$

where *G* represents the intensity in green channel after median filtering.

Next, *d*_2_ is dilated with circle structuring element *e*_3_ as expressed in

(7) $$\begin{array}{c} d_{2}=d_{2}⊕e_{3}. \end{array}$$

A subtraction operation is performed between *d*_1_ and *d*_2_ to enhance the boundary of bright lesions:

(8) $$\begin{array}{c} D=d_{2}-d_{1}. \end{array}$$

**Figure 6:**
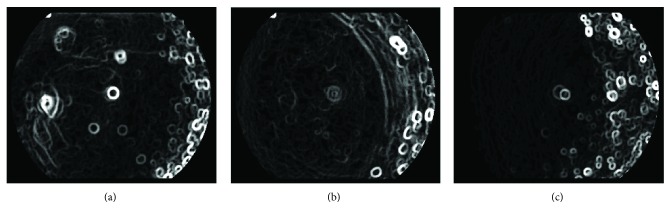
The enhancement of vitreous opacity. (a), (b), and (c) correspond to the original images in Figures 2(a), 2(b), and 2(c), respectively.

Equations (5) and (6) aim to erode dark vessels, so the selection of the size of circle structuring elements is based on the width of the main retinal vessels. In our experiments, the main vessels' width of normalized image is in the range of 24∼36 pixels. Accordingly, the radius of *e*_1_ and *e*_2_ is set to 24 and 36, respectively. The radius of *e*_3_ decides the width of the detected boundaries of the candidate vitreous opacity, and the width should be slightly wider than the average width of the vessels. Through experiments, it is suitable to set *e*_3_ to 28. Figures 6(a), 6(b), and 6(c) show the enhancement of vitreous opacity, which correspond to the original images in Figures 2(a), 2(b), and 2(c), respectively.

By thresholding in image *D*, boundaries of bright regions will be detected as candidate vitreous opacity, which contain the boundaries of vitreous opacity, the optic disc, and few vessels across the optic disc. To judge whether there is vitreous opacity in an input image, thresholding is performed on image *D* according to (9). The number of segmented pixels and number of connected regions are computed. Through testing, when *t*_*d*_ is set to 8, vitreous opacity in most images can be detected well in our database. If the number of segmented pixels is much larger than that of the optic disc perimeter or if the number of connected domains is more than 5, it is considered that there is possible vitreous opacity in this image.

(9) $$\begin{array}{c} D_{t}\left(x,y\right)=\left\{\begin{array}{cc} 1, & D\left(x,y\right)\geq t_{d}\\ 0, & D\left(x,y\right)<t_{d} \end{array}\right.. \end{array}$$

As shown in Figure 6(a), when the optic disc is obvious in retinal images, the boundary of the optic disc and vessels around the optic disc will be enhanced in image *D* as well. Therefore, the region around the optic disc should be excluded from vitreous opacity detection. A method of optic disc removal is proposed and applied only on the images with possible vitreous opacity.

Because the optic disc is usually the largest bright region in a retinal image, the 2% brightest pixels in the ROI are selected as candidate pixels of the optic disc. If *B* is defined as the selected bright pixels, a clustering algorithm is applied on *B* to cluster these pixels into groups based on Euclidean distance. The candidate optic disc is searched among these groups according to the size of the optic disc.

Features in image *D*_*t*_ are taken into consideration to search the optic disc. For group_*i*_, the number of pixels in *D*_*t*_ is summed up in a window *W* centered on the center of group_*i*_ as follows:

(10) $$\begin{array}{c} sum\_p=\underset{\left(x,y\right)\in W}{\sum }D_{t}\left(x,y\right). \end{array}$$

If sum_*p* is larger than *p*_2_, then this group is considered as the optic disc. Then, detected candidate vitreous opacities in the square with the size 1.5  optic  disc  diameter × optic  disc  diameter centered on the center of the optic disc are excluded. If there is no region satisfying the above criteria, it is considered that there is no visible optic disc in the image. The whole process is expressed as in Algorithm 1.

The parameters *p*_1_, *p*_2_, and *l* are all determined by the size of the optic disc. The optic disc is roughly an ellipse and its long diameter is about 200 pixels in our experiments. The bright pixels with the distance less than *l* are all gathered into one region, and *l* is set to be the diameter of the optic disc. *p*_1_ is utilized to judge whether the clustered group is a candidate of the optic disc. Considering that not all pixels in the optic disc can be selected as the brightest pixels, *p*_1_ is set to 2/3 × area  of  the  optic  disc. sum_*p* is the area of detected edges in window *W*. *W* should cover the optic disc, which is set to 1.5  optic disc  diameter × 1.5 optic  disc  diameter. *p*_2_ is employed to detect whether the clustered region contains the edges of the optic disc. *p*_2_ should be at least larger than the area of optic disc boundaries. Because the width of disc edges is close to the vessel width, *p*_2_ is set to vessel width × optic  disc  perimeter.

### 2.4. Feature Extraction

It is important to extract appropriate features from a retinal image for classifying cataract.  Figure 1 illustrates that retinal structures that are clearer and can be seen in images with lower grades. According to the grading protocol mentioned in Section 1, the contrast between retinal structures and background is the prime feature for grading. Considering the above, three types of features which contain nine features are selected in this paper. These nine features are described in Table 1.

#### 2.4.1. Visible Retinal Structure Segmentation Using Multiple Thresholds

**Figure 7:**
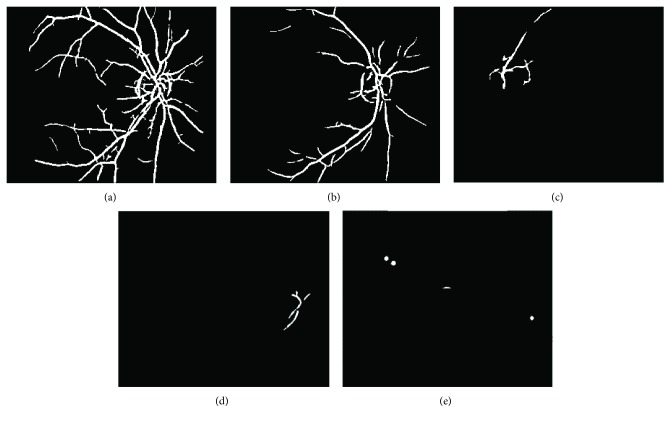
Visible structure segmentation. (a), (b), (c), (d), and (e) correspond to the original images in Figures 1(a), 1(b), 1(c), 1(d), and 1(e), respectively.

Vessels and boundary of the optic disc are enhanced with the Gaussian-matched filter as described in Section 2.2. More obvious structure has higher response. Retinal structure pixels are segmented by thresholding the enhanced image *E*_image(*x*, *y*) with a given threshold value *t*_*i*_, and a visible structure map *V*_*i*_ can be generated as

(11) $$\begin{array}{c} V_{i}=\left\{\begin{array}{ll} 1, & if\;E\_image\left(x,y\right)\geq t_{i}\\ 0, & if\;E\_image\left(x,y\right)<t_{i} \end{array}\right., \end{array}$$

where (*x*, *y*) is the position of every pixel in the image and *V*_*i*_ is a binary image in which visible structure pixels are expressed as 1.  Figure 7 shows some examples of visible structure results with different grades of cataract by setting *t*_*i*_ to 0.9. The images in Figure 7 correspond to the original images in Figures 1(a), 1(b), 1(c), 1(d), and 1(e), respectively. It is evident that there is the largest number of visible structure pixels in a healthy image, and the number of segmented pixels can describe the severity of cataract. The number of visible structure pixels *v*_*i*_ is computed as follows:

(12) $$\begin{array}{c} v_{i}=\sum_{\left(x,y\right)\in V_{i}}V_{i}\left(x,y\right). \end{array}$$

High threshold will result in missing vessels in the retinal images with serious blur, but on the other hand, low threshold will produce noises in the retinal images with lower grades. Visible structure segmentation with only one threshold is not enough to classify retinal images into 5 different grades accurately. Therefore, four increasing threshold values *t*_1_, *t*_2_, *t*_3_, and *t*_4_ are applied using (11) separately, and each threshold is only employed to distinguish two adjacent grades. *t*_1_ is set to classify grades 3 and 4, *t*_2_ is for the classification of grade 2 and grade 3, and so on. The value *t*_1_ should not be too low to avoid over segmenting nonvessel pixels in grade 3 and grade 4. The value *t*_4_ should not be set too high to detect small vessels in the retinal images with grade 0. With the blurriness aggravating, the difference between two adjacent grades is less. Accordingly, difference between the two adjacent thresholds is increasing. Through experiments of sample images from five grades, *t*_1_, *t*_2_, *t*_3_, and *t*_4_ are set to 0.3, 0.5, 0.9, and 1.4, respectively, in this paper. Four visible retinal structure segmentation *V*_1_, *V*_2_, *V*_3_, and *V*_4_ are obtained, which correspond to the four thresholds *t*_1_, *t*_2_, *t*_3_, and *t*_4_. Then, the number of segmented pixels in *V*_1_, *V*_2_, *V*_3_, and *V*_4_ is summed and denoted as *v*_1_, *v*_2_, *v*_3_, and *v*_4_, respectively, via (12), which is described in Table 1.

As mentioned in Section 2.3, responses of the 2D Gaussian filter for the pixels around vitreous opacity are strong, which will be detected as visible retinal structure pixels (see Figures 9(a-1), 9(b-1), and 9(c-1)). If an input image is diagnosed as vitreous opacity, boundaries of vitreous opacity should be eliminated from segmentation results *V*_1_, *V*_2_, *V*_3_, and *V*_4_.

It is hard to eliminate vitreous opacity directly from visible structure segmentation due to the large variation among the images with different blur levels. Input images are classified into two classes: class 1 containing images with visible retinal structures (grades 0, 1, and 2) and class 2 including images with invisible retinal structures (grades 3 and 4). Vitreous opacity is removed from visible structure segmentation using different algorithms for these two classes.

**Figure 8:**
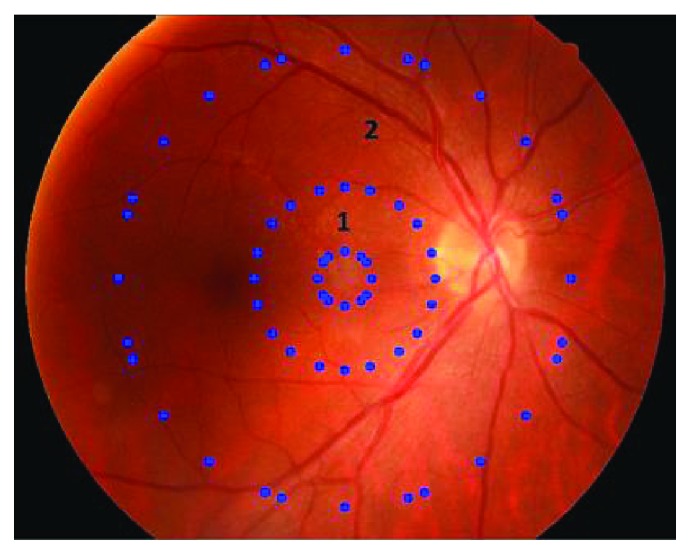
The partition of a retinal image.

For this rough classification, a retinal image is divided into two annular regions: region 1 and region 2 (refer to Figure 8). Average enhanced values of *E*_1_ and *E*_2_ using the Gaussian filter in these two regions are computed as follows:

(13) $$\begin{array}{c} E_{1}=\frac{1}{n_{1}}\sum_{\left(x,y\right)\in R_{1}}E\_image\left(x,y\right), \end{array}$$

(14) $$\begin{array}{c} E_{2}=\frac{1}{n_{2}}\sum_{\left(x,y\right)\in R_{2}}E\_image\left(x,y\right), \end{array}$$

where *R*_1_ and *R*_2_ represent region 1 and region 2, respectively. *R*_1_ contains no retinal structures and *R*_2_ contains the main structures. And *n*_1_ and *n*_2_ are the number of pixels in *R*_1_ and *R*_2_ correspondingly. *E*_image is the enhanced image with Gaussian filter correlation.

Average values in two regions are added up as follows:

(15) $$\begin{array}{c} E=E_{1}+E_{2}. \end{array}$$

According to the value *E*, an input image is categorized into two classes by thresholding:

(16) $$\begin{array}{c} \left\{\begin{array}{c} E\geq t_{e},\;input\in class\;1\\ E<t_{e},\;input\in class\;2 \end{array}\right., \end{array}$$

where *t*_*e*_ is a value that is trained using bipartition [38] in the sample set.

To eliminate vitreous opacities from four different vessel maps, *V*_1_, *V*_2_, *V*_3_, and *V*_4_, boundaries of vitreous opacity are extracted by applying three thresholds on image *D* via

(17) $$\begin{array}{c} D_{i}\left(x,y\right)=\left\{\begin{array}{cc} 1, & if\;D\left(x,y\right)\geq {td}_{i}\\ 0, & if\;D\left(x,y\right)<{td}_{i} \end{array},i=1,2,3\right., \end{array}$$

where *td*_1_ < *td*_2_ < *td*_3_. In this paper, *td*_1_, *td*_2_, and  *td*_3_ are set to 2, 5, and 8, respectively, via experiments. The extracted results are expressed as binary images *D*_1_, *D*_2_, and  *D*_3_ in turn and are further expanded using circle structuring element *e*_4_ as follows:

(18) $$\begin{array}{c} D_{i}=D_{i}⊕e_{4}. \end{array}$$

The radius of *e*_4_ is set to 5 pixels to ensure that the width of vitreous opacity boundary is wider than the vessel diameter.

It is observed that most vitreous opacities are obvious in the retinal images of class 1, but a lot of vitreous opacities are dim in retinal images of class 2. Due to higher contrast of the images in class 1, many nonvitreous opacity pixels are detected in *D*_1_  and  *D*_2_. In the images of class 2, most vitreous opacities can be detected well in *D*_1_  and  *D*_2_, but many dim vitreous opacities will be missed in *D*_3_. Therefore, different algorithms for class 1 and class 2 are proposed to remove vitreous opacities from *V*_1_, *V*_2_, *V*_3_, and *V*_4_. Due to the strong contrast between vitreous opacities, some images with large number of vitreous opacities of class 2 may be misclassified as class 1. So for images in class 1, if there are still many connected regions with small areas in *V*_1_ after removal of vitreous opacities, it is considered that this image is misclassified as class 1 and further elimination needs to be performed on *V*_1_ and *V*_2_. The whole process of eliminating vitreous opacity is as in Algorithm 2.

**α** is a set of connected regions in *V*_1_, and *N* is the number regions in **α**. Because single vitreous opacity is a region of a small area, **β** is the set of connected regions with a small area that is less than half of the area of the optic disc and *K* is the number of regions in **β**.

**Figure 9:**
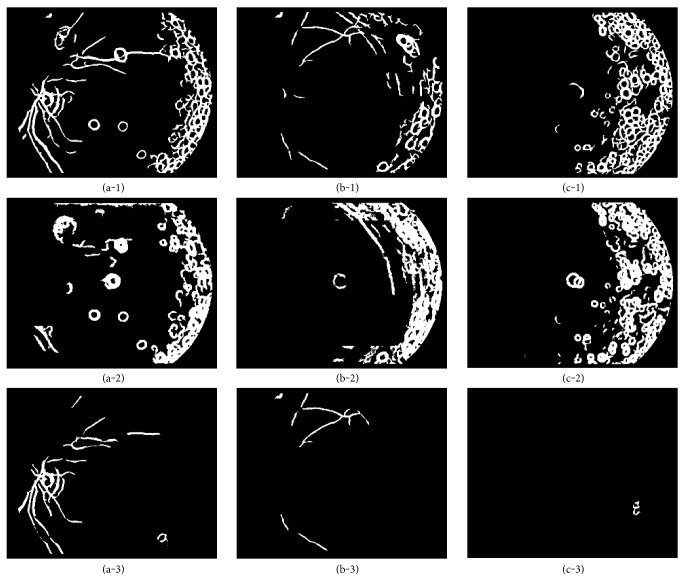
Examples of vitreous opacity removal from visible retinal structure segmentation. The first row is the segmented visible structures in three retinal images without removing vitreous opacity. The second row shows the extracted boundaries of vitreous opacity. The last row is the final visible structure detection after removing vitreous opacity boundaries. The images in columns 1, 2, and 3 correspond to the original image in Figures 2(a), 2(b), and 2(c), respectively.

Examples of vitreous opacity boundary removal from visible retinal structure segmentation are shown in Figure 9. Final visible retinal structure maps are illustrated in Figures 9(a-3), 9(b-3), and 9(c-3)), and Figures 9(a-2), 9(b-2), and 9(c-2)) is the extracted vitreous opacity boundaries. It can be noted from Figure 9 that the proposed method can detect vitreous opacity well in these images and the extraction of retinal feature is more accurate after vitreous opacity removal.

#### 2.4.2. Mean Gaussian Filter Response of Segmented Visible Retinal Structure Pixels

The value of a pixel in the enhanced image implies the contrast in the original image. Mean enhanced value of segmented pixels is described as follows:

(19) $$\begin{array}{c} m_{i}=\frac{1}{S_{num}}\sum_{\left(x,y\right)\in s}E\_image\left(x,y\right). \end{array}$$

In (19), *S* is a group of the segmented vessel pixels with a specified threshold value. *E*_image(*x*, *y*) represents the value of the point (*x*, *y*) in the enhanced image. *s*_num_ denotes the number of all segmented vessel pixels. Four mean enhanced values (*m*_1_, *m*_2_, *m*_3_, and *m*_4_ presented in Table 1) are computed with four different thresholds.

#### 2.4.3. Local Standard Deviation

A clearer image owns a sharper contrast, which corresponds to greater intensity variance. Accordingly, local variance is computed on every pixel within the ROI mask. The neighborhood standard deviation is calculated within the *n* × *n* neighborhoods around the corresponding pixels. Mean value of the local standard deviations *d* (presented in Table 1) in the ROI mask is calculated as a feature. The value of *n* can be set in a range of 0∼vessel width. Large value of *n* will lead to long computing time. Experiments show that different values of *n* will not affect the classification results too much. To save computing time, *n* is set to 3 in this paper.

### 2.5. Grading of Blurriness

**Figure 10:**
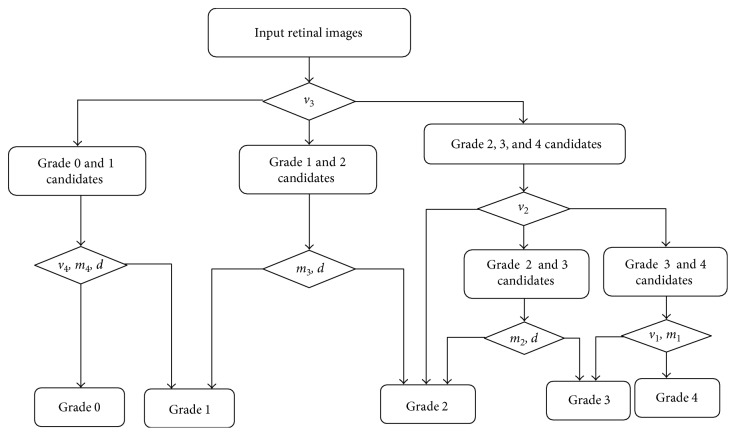
The diagram of classification.

Because the distribution of features overlaps between neighboring grades, a decision tree is trained to classify a retinal image according to the blurriness. A sample set *S* selected from database is used to train grading parameters. The whole process is demonstrated in Figure 10.

Firstly, feature *v*_3_ is selected to classify input images into three classes: a group of images with grades 0 and 1, a group of images with grade 1 and grade 2, and a group of images with grade 2, grade 3, or grade 4. It can be described as follows:

(20) $$\begin{array}{c} \begin{array}{ll} v_{3}<s-\Delta s; & input\in grades\;0\;and\;1\\ -\Delta s\leq v_{3}\leq \Delta s; & input\in grade\;1\text{ or }2\\ v_{3}>s+\Delta s; & input\in grades\;2,3,and\;4. \end{array} \end{array}$$

Here, the interval of feature *v*_3_ in the overlapped distribution between sample images in grade 1 and grade 2 is defined as an increasing order {*s*^1^, *s*^2^, ..., *s*^*n*^}. *s* is the average value of feature *v*_3_ in the overlapped distribution between sample images in grade 1 and grade 2. Δ*s* is computed as follows:

(21) $$\begin{array}{c} \Delta s=\frac{\left(s^{1}+s^{n}\right)}{2}. \end{array}$$

Next, feature *v*_2_ is used to classify the retinal images into three classes similarly as feature *v*_3_. Finally, images are graded into five different grades according to different features as Figure 10 shows. As retinal structures are hard to be seen in the images with the cataract in grades 3 and 4, feature *d* is not used for classification between grades 3 and 4.

For classifying two neighboring grades (grade *i* and grade *i +* 1), threshold *T*_*i*_ of the employed feature is trained using bipartition proposed by Quinlan [38]. Information gain of the feature *f*_*i*_ based on threshold *T*_*i*_ is calculated as follows:

(22) $$\begin{array}{c} Gain\left(S,f_{i}\right)=Ent\left(S\right)-\underset{\lambda \in \left\{-,+\right\}}{\sum }\frac{\left|S_{T}^{\lambda }\right|}{\left|S\right|}Ent\left(S_{T}^{\lambda }\right), \end{array}$$

where *S* is the sample set, *S*_*T*_^−^ is the sample set in which *f*_*i*_ is less than threshold *T*_*i*_, and *S*_*T*_^+^ is the sample set in which *f*_*i*_ is larger than *T*_*i*_.

Ent is the information entropy, which can be expressed as follows:

(23) $$\begin{array}{c} Ent\left(S\right)=-\sum_{k=1}^{N}p_{k}{log}_{2}p_{k}, \end{array}$$

where *p*_*k*_ is the proportion of class *k* in the sample set and *N* is the number of the employed features.

A voting scheme is applied for final grading, and the voting score is calculated as follows:

(24) $$\begin{array}{c} score=\sum_{i=1}^{N}v_{f_{i}} \end{array}$$

and

(25) $$\begin{array}{c} v_{f_{i}}=\left\{\begin{array}{ll} 1, & if\;f_{i}\geq T_{i}\\ 0, & if\;f_{i}{<T}_{i} \end{array}\right.. \end{array}$$

If the score is larger than *N*/2, the image is classified into grade *i*. Otherwise, the grade is decided by *t*, the feature with the maximum information gain.

## 3. Results and Discussion

### 3.1. Data Description

In our experiment, retinal images from the Beijing Institute of Ophthalmology, Tongren Hospital, are tested to evaluate the proposed grading algorithm. This database consists of images with different resolutions which include 1396 × 1124, 1572 × 1308, 1704 × 1496, 2036 × 1696, 2196 × 1740, 3048 × 2432, and 3380 × 2592 pixels. In total, 1355 retinal images of the right or left eyes from more than 1000 patients (all are Chinese) aged 20–80 in China were tested. In this database, most are 45-degree retinal images which are centered on the macula and only 15 retinal images are centered on the optic disc. These retinal images are graded by two experienced ophthalmologists according to the criterion [10] described in Section 1. Our automatic grading is compared with this clinical grading.

### 3.2. Experimental Results and Discussion

In total, 1355 retinal images are collected to evaluate our automatic blurriness grading system and the dataset includes 433, 415, 217, 133, and 157 images with blur level in grade 0, grade 1, grade 2, grade 3, and grade 4, respectively. The evaluation criteria and clinical diagnosis are presented in Table 2. The five-fold cross-validation method is used to evaluate the performance of our proposed method. The retinal images of our database are randomly divided into five subsets. In each fold, one subset is chosen as a testing set and the other four subsets are the training data.

The results of all folds are counted. The joint accuracy for all folds of different types of clinical evaluation compared with that of manual grading are shown in Table 3, and confusion matrix of five gradings is presented in Table 4. Compared with that of the clinical grading, the joint accuracy of five-grade grading can reach 81.1%. Screening between noncataract and cataract using the proposed algorithms can achieve an accuracy of 92.8%; specific performance metrics of recognizing cataract are described in Table 5. In Table 5, TP, FN, FP, and TN represent true positives, false negatives, false positives, and true negatives, respectively. According to the grading criteria [13, 35], the images in grade 0 are considered noncataract, while the images in grades 1 and 2 correspond to mild and moderate cataract, respectively. The rest of the images in grades 3 and 4 are classified into severe cataract. The accuracy reached 83.8% when retinal images are classified into 4 grades (healthy, mild, moderate, and severe) [13, 35]. Blurriness in grade 1 will not cause severe vision loss, while blurriness in grades 2, 3, and 4 will lead to blindness which needs timely surgery [11]. Hence, it is important to recognize those images that need surgery. Ophthalmologists suggest that classification between grade 0 and grade 1 will not influence surgical screening. After neglecting the classification between grades 0 and 1, the accuracy compared with that of manual grading is 88.4%.

By taking clinical grading as the gold standard, the agreement between the automatic cataract grading system and clinical grading in five-grade grading is measured by kappa statistic. Kappa coefficient is a statistic which measures inter-rater agreement for qualitative items [39]. It is applied extensively in clinical studies [40] and clinical skill assessments [41]. The formula for Cohen's kappa coefficient is expressed as follows:

(26) $$\begin{array}{c} Kappa=\frac{p_{o}-p_{e}}{1-p_{e}}, \end{array}$$

where *p*_*o*_ is the observed percentage of agreement which implies the proportion of ratings where the raters agree and *p*_*e*_ is the expected percentage of agreement which denotes the proportion of agreements that are expected to occur by chance as a result of random rating.

The range of kappa is from 0∼1, with larger values indicating better agreement. If the kappa value is greater than 0.61, a study shows that such a classification algorithm is an applicable method for grading [42].  Table 6 shows that our kappa value is 0.7435 and variance and *P* value are both less than 0.001. It indicates that there is no large deviation between automatic grading and clinical grading.

Vitreous opacity is a common disease which will influence feature extraction and grading of blurriness. As seen in Table 7, 23 retinal images are seriously influenced by this type of inaccurate feature extraction and their grading difference is more than two grades. A morphological method with multithresholds is proposed to extract and remove lesions such as vitreous opacity. Large grading difference is thus eliminated; 4.4% improvement made is achieved for five-grade grading of blurriness.

To compare with other algorithms of cataract classification using retinal images, we have implemented these algorithms and tested these algorithms using our database. The comparison results are summarized in Table 8. Due to small differences between adjacent grades, the Fourier score proposed in paper [35] describes that the high-frequency components of an input image cannot achieve a promising result in cataract screening or grading. Forty features are extracted in the method [36] to classify the cataract without considering other pathologies. Experimental results show that there are large grading differences using this method [36]. For example, maximal deviation by this method [36] is more than 3 grades. Compared with that of these methods, the proposed algorithm achieved more promising results and performed well in processing images with pathological changes.

**Figure 11:**
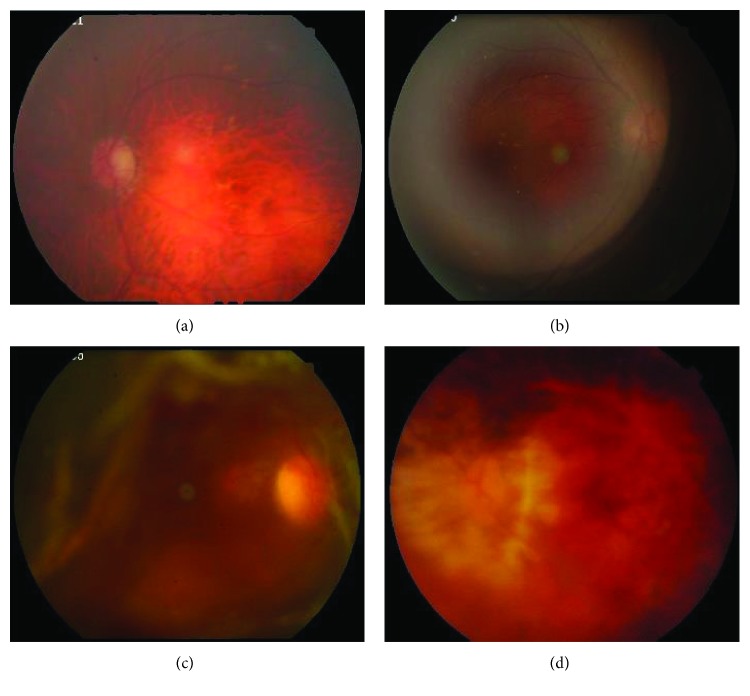
Analysis of the grading difference.

Analyzing the grading difference, we found that the proposed algorithm still has limitation in handling images with the following cases: uneven illumination, blurriness of the retina due to the small pupil, or large area of lesions.  Figure 11 shows some examples of such cases. Figures 11(a) and 11(b) display the retinal images with uneven illumination. In Figure 11(a), the blur of the retina is due to patient's small pupil, so ophthalmologists diagnosed this image as cataract in grade 1, while our automatic system considered it as cataract in grade 2. The uneven illumination of Figure 11(b) is caused by the operation of taking the picture. The small vessel can be seen in the bright region, but it is invisible in the dark area. This image is considered cataract in grade 1 in clinical grading while it is graded into grade 2 by the automatic system. Figures 11(c) and 11(d) show the examples of retinal blurriness caused by other lesions, so ophthalmologists tend to classify these images into lighter grades. As Figure 11(c) shows, the lesions cover the main retinal structures. Our algorithm classified Figure 11(c) as cataract in grade 3, but ophthalmologists think it is in grade 2. The blurriness of Figure 11(d) is caused by high myopia, so grade 2 is determined by ophthalmologists and grade 3 is obtained using our grading system.

Different retinal diseases may appear on a retinal image, such as macular edema, diabetic retinopathy, peripapillary atrophy, and vitreous opacity. In our tested database, most images only have cataract and parts of retinal images have other pathologies, such as mild diabetic retinopathy and vitreous opacity. Our proposed method performs well in grading cataract in these images. However, because a large area of severe pathologies will affect the detection of retinal structures, grading difference also exists among different ophthalmologists. Therefore, cataract screening of retinal images with severe pathologies is still a challenge for manual grading as well as automatic grading. Our future work will focus on handling the retinal images with complicated pathologies. Moreover, there are causes of blur other than cataract, such as media opacities in the anterior segment (e.g., cornea opacities) or posterior segment (e.g., vitreous haemorrhage), the small pupil, and process of photo taking. The small pupil of patients will cause blur of retinal images, but it mostly affects the peripheral regions of the retinal images. Generally, the retinal images of patients will be photographed with the mode of the small pupil. For blurriness caused by photo taking, these images are taken under strict protocol and images with bad quality will be taken again. At the current stage of research, our proposed method cannot recognize the causes of blurriness yet. The blur images caused by other media opacities may affect the grading of cataract. Identifying the cause of blurriness will be investigated in our future work. There are still some parameters that are currently set empirically, which may not be applicable to retinal images with different fields of view or other ethnicities. In the future work, automatic estimation of the parameters will be studied to improve the generalization of the proposed approach.

## 4. Conclusion

An approach to evaluate blurriness with vitreous opacity removal for cataract diagnosis using retinal images is proposed. Three types of features based on image enhancement using the 2D Gaussian filter are extracted to describe the blurriness in a retinal image. A decision tree is further proposed to classify the image into five grades. Compared with other research works in cataract grading using a retinal image, it is the first time that detection and removal of pathological changes are used to improve the grading accuracy. Using a large amount of clinical images as test data, our system can obtain 81.1% accuracy of exact cataract grading and 92.8% accuracy of two-class screening (noncataract and cataract). Kappa value is 0.7435 and variance and *P* value are both less than 0.001 in the five-grade grading, which illustrates that there are no large differences between our grading and manual grading. The proposed approach provides an accurate and universal measure of cataract severity using a retinal image. The proposed system is currently in the stage of clinical validation, and it can be applied to mass screening and remote consultation in the future.

## Figures and Tables

**Algorithm 1:**
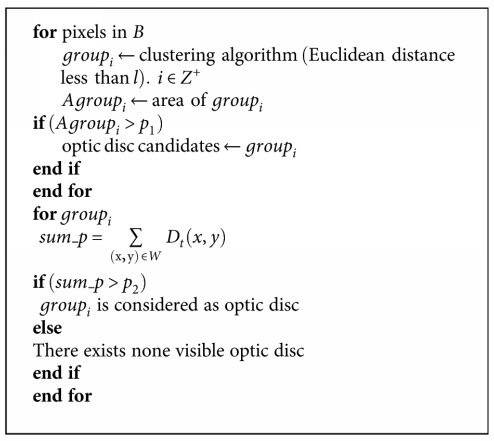


**Algorithm 2:**
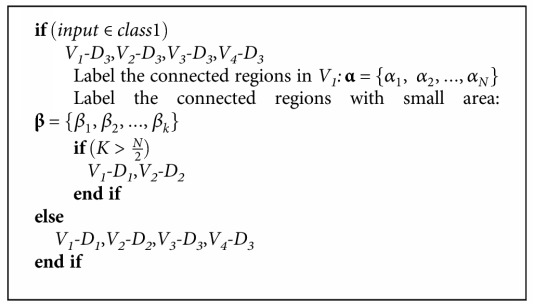


**Table 1:** Feature description of cataract grading.

Feature parameter	Description
*v*_1_	Number of visible retinal structure pixels segmented with threshold *t*_1_
*v*_2_	Number of visible retinal structure pixels segmented with threshold *t*_2_
*v*_3_	Number of visible retinal structure pixels segmented with threshold *t*_3_
*v*_4_	Number of visible retinal structure pixels segmented with threshold *t*_4_
*m*_1_	Mean enhanced value of segmented visible retinal structures with threshold *t*_1_
*m*_2_	Mean enhanced value of segmented visible retinal structures with threshold *t*_2_
*m*_3_	Mean enhanced value of segmented visible retinal structures with threshold *t*_3_
*m*_4_	Mean enhanced value of segmented visible retinal structures with threshold *t*_4_
*d*	Average value of the local standard deviation

**Table 2:** Evaluation criteria.

Evaluation of grading	Definition
Two-class classification (noncataract/cataract)	Noncataract: grade 0
	Cataract: grades 1–4
Surgery indicator	No surgery: grades 0-1
	Surgery class 1: grade 2
	Surgery class 2: grade 3
	Surgery class 3: grade 4
Four-grade grading [13, 36]	Noncataract: grade 0
	Mild cataract: grade 1
	Moderate cataract: grade 2
	Severe cataract: grades 3-4
Five-grade grading of blurriness [10]	Grades 0, 1, 2, 3, and 4 (refer to Figure 1)

**Table 3:** Results of automatic grading using different types of clinical evaluation.

	Two-class classification (noncataract and cataract)	Surgery indicator (combining grades 0 and 1)	Four-grade grading	Five-grade grading of blurriness
Joint accuracy	92.8%	88.4%	83.8%	81.1%

**Table 4:** Confusion matrix of grading.

Graders' grades	Automated grades				
	0	1	2	3	4
0	399	34	0	0	0
1	64	314	37	0	0
2	0	33	146	38	0
3	0	0	13	93	27
4	0	0	0	9	148

**Table 5:** Performance of recognizing cataract.

Measure		Description
TPR	0.931	TP/number of cataract images
FPR	0.785	FP/number of noncataract images
Specificity	0.921	TN/(TN + FP)
Sensitivity	0.931	TP/(TP + FN)
Accuracy	0.928	(TP + TN)/number of all images

**Table 6:** Statistics of validation experiment in five-grade grading.

	Cohen's kappa	Kappa error	Variance	*P* value
Test set (1355 images)	0.7453	0.0142	0.0002	<0.001

**Table 7:** Comparison results with vitreous opacity removal.

	Surgery indicator (combining grades 0 and 1)	Grading criteria in 4 grades	Five-grade grading of blurriness		
			Difference ≥ 2 grades	Difference = 1 grade	Accuracy
Grading without improvement	84.8%	80.8%	23	293	76.7%
Grading with removing vitreous opacity	88.4%	83.8%	0	255	81.1%

**Table 8:** Performance comparison with other methods.

Technique	Two-class classification (noncataract and cataract)	Four-grade grading of cataract	Five-grade grading of blurriness	Difference ≥ 2 grades
Abdul-Rahman et al. [35]	61.9%	—	—	—
Yang et al. [36]	90.1%	77.4%	72.3%	14
Proposed method	92.8%	83.8%	81.1%	0
